# Dealing with disruptions in railway track inspection using risk-based machine learning

**DOI:** 10.1038/s41598-023-28866-9

**Published:** 2023-02-07

**Authors:** Sakdirat Kaewunruen, Mohd Haniff Osman

**Affiliations:** 1 Department of Civil Engineering, University of Birmingham, Birmingham, B15 2TT UK; 2 Sustainable Urban Transport Research Centre, Faculty of Engineering and Built Environment, The National University of Malaysia, 43600 UKM Bangi, Malaysia; 3 Department of Engineering Education, Faculty of Engineering and Built Environment, The National University of Malaysia, 43600 UKM Bangi, Malaysia

**Keywords:** Civil engineering, Mechanical engineering, Natural hazards

## Abstract

Unplanned track inspections can be a direct consequence of any disruption to the operation of on-board track geometry monitoring activities. A novel response strategy to enhance the value of the information for supplementary track measurements is thus established to construct a data generation model. In this model, artificial (synthetic) data is assigned on each measurement point along the affected track segment over a short period of time. To effectively generate artificial track measurement data, this study proposes a NARX (nonlinear autoregressive with exogenous variables) model, which incorporates short-range memory dependencies in the dependent variable and integrates interdependent effects from external factors. Nonlinearities in the proposed model have been determined using an artificial neural network that allowed fast computation of a mapping function in line with the needs of effective disruption management. The risk of over fitting the data generation model, which reflected its generalisation ability, has been effectively managed through risk aversion concept. For the model evaluation, the deviation of track longitudinal level has been taken as a case study, predicted using its degradation rate and track alignment and gauge as exogenous variables. Simulation results on two datasets that are statistically different showed that the data generation model for disrupted track measurements is reliable, accurate, and easy-to-use. This novel model is an essential breakthrough in railway track integrity prediction and resilient operation management.

## Introduction

Geometric conditions of a railway track are measured and updated regularly through an assessment of track geometry measurements collected by a track recording car integrated with inspection data from visual inspections. These examinations ensure running safety and ride comfort of rolling stocks on a railway network. Before a service failure, defective tracks receive appropriate track maintenance activities (e.g. tamping, rail resurfacing, reconditioning, etc.) subject to the track maintenance policy, either in line with corrective or preventive measures. Requisite for an effective maintenance operation is a track measurement/inspection schedule (TIS). To date, TIS is modelled as an optimisation problem, which incorporates various temporal and spatial parameters, as well as physical (i.e. human and machine) characteristics^1^. As the TIS is exposed to uncertainties in a real-time environment, disruptions can occur and potentially offset some or all of the remaining activities in the schedule^1,2^.

Disruption is a random event and not a deterministic process, and this characteristic makes its presence unpredictable and dynamic. When disruption to the TIS incurs, we could expect a delay in supplying track measurements and inspection data informing track irregularity to the planning process of track maintenance. Leaving tracks unattended longer than necessary increases the risk of late defect detection. As a result, unplanned maintenance instead of a planned function must be performed. Certainly, this has a direct impact on track maintenance expenses^3^. In an extreme situation, ineffective track inspection could become a causal factor of train derailment^4,5^. One possible way to respond to a disruptive event in track inspection is by finding an appropriate response action to disrupted track measurement. The term ‘disrupted’ is used to emphasise a direct loss attributed to disruption in TIS. In this study, an innovative framework of disruption management (DM) has been employed to identify an appropriate response action for disrupted TIS.

The motivation of employing the DM framework is that a response action can impose the minimum change possible in all aspects of the affected process or system of interest. A smooth transition from pre- to post-disruption is required because disruption is temporary in nature. To satisfy this requirement, a system engineer must know and preserve as much as possible the integrity of the ‘disrupted’ component or unit of the process^6^. In this study, track measurement data serves as the representative information of ‘disrupted’ component.

A railway track is an example of a complex and continuous asset; thus, its length is a critical parameter in the track maintenance equation. This description means that from a statistical point of view, track measurements are spatially correlated within a certain interval. This dependency could introduce a bias in an analysis of data model identification. Similarly, periodic track measurements provide a platform to trace an evolution of track geometry changes over time (tonnage). Hence, both temporal and spatial effects should be considered when deciding upon an appropriate response action for disrupted track measurements. In line with the requirements above, an application of artificial intelligence to derive adaptive responses to a disrupted TIS appears to be promising. We therefore propose a novel artificial intelligence framework to predict track geometry deterioration. Briefly stated, the new method first constructs a statistical model for track measurement data before being used to predict the missing track measurement data.

The main goal of developing a prediction model is to provide an approximation to the actual physical process or system and predict its outputs^7^. When a thorough nonlinear relationship between input covariates and the model output of the process is not necessary, statistical models are more effective for the creation of prediction models. This method uses a simplified mathematical function to describe dynamic interactions in the input/output variables^8^. One of the most convenient model structures for prediction purposes is the nonlinear autoregressive model with exogenous variables (NARX). In this model, a nonlinear mapping function defines the complex relationship between the targeted (output) and external (input) variable. The function takes past values of the input and output variables to generate the current value of the output variable(s). In relation to the method used to obtain the mapping function, the NARX-NN model could have different shapes of function. Among various methods such as Box-Jenkins model, neural networks have been proven to be universal function approximators, that is, it can approximate complex functions with arbitrary accuracy conditioned by the number of training epochs and quality of data^9^.

Neural networks have become popular in simulation and prediction for two reasons; they handle the nonlinear relationship between input–output variables implicitly (i.e. without requiring an in-depth knowledge or making assumptions about the problem under investigation), and they generalise well against unseen data. However, the beneficial features of a neural network would disappear if it is trained using a small set of training data. A lack of data can occur in the case of disrupted track inspection, particularly for track sections associated with low measurement frequency which thereafter are prone to train derailment^10^. Ones should note that only measurements recorded from the last tamping maintenance (i.e. a restoration point) will be used for modelling. Any track measurements gathered outside from the interval is declared obsolete because the state of an analysed track has been reset by maintenance work. This restriction placed on data preparation for the neural network is unique for TIS. In addition, safety-based maintenance has been discussed to optimise geometry restoration of railway systems facing uncertainties and disruptions. Bayesian network-based approaches have been adopted to evaluate effects of weather on component failures at railway turnouts (RTs)^11,12^. However, the study was not applicable to large-scale investigations as climate pattern often varies. The method has some limitation since it requires the data to be segmented according to climate patterns, failure conditions, and spatial data within a given time.

Overestimation is one of the side effects of overfitted data. To mitigate the overestimation risk in prediction models, a neural network utilises Bayesian regularisation. Bayesian regularisation limits the size of the network parameters to make the network produce a smoother response. Apart from its strong generalisation properties, a regularised neural network also takes less computational time for model training. The model validation process, which is scaled as $\mathrm{\rm O}\left({N}^{2}\right)$ where *N* is the number of data points, is no longer necessary^13^. The time-saving benefit appears in the case of large quantities of data.

To date, several predictive models have been proposed^14–16^, but all models are not designed with respect to the unique nature of disruptions to track inspection. To that end, an integrated formulation of a Bayesian regularised neural network and the NARX model best fits to generate AI-based data for track measurements in the presence of unexpected events. In this paper, the selected track geometrical parameter is defined as an autoregressive variable, while the other parameters act as external inputs in the NARX model. The selected parameter has a significant role in the track maintenance decision^17^. Since a neural network is driven by data, this study applies machine learning tools such as clustering in the proposed model to adopt risk aversion concept, in order to generate a quality training–testing data set. This action significantly reduces a neural network’s computational time. Overall, the promising results reported in this study illustrate the importance of formulating an efficient method of fusion to construct a prediction model that generalises well to new instances, as reflected in the accuracy of the predicted results.

## Method

### Formulation of the prediction model

During a track geometry measurement, the track recording car (TRC) travels at a track speed of between 70 and 120 km/h to measure seven track geometrical parameters at equally-spaced track points (positions) over a single track code. One track code spans *K* kilometres of track and is thus divided into several non-overlapping track segments. Note that *K* is the unit length of track route in kilometres. For a track segment *S* with a length of *m* metres (note that *m* is the unit length of track segment in metres), there can thus be a sequence of measurement points, denoted as where $i=\mathrm{1,2},\dots ,m/r$ and . Here, *r* denotes the distance (in meters) between two adjacent measurement points. When the *n*th track measurement is carried out in track segment , each ${x}_{i}^{{S}_{j}}$ for is assigned with an amount of deviation of *G* track geometrical parameters. Here, when subscript k refers to track condition index, it is expressed as wherein $k=\mathrm{1,2},\dots ,G$.

When a disruption incurs, for example in the TRC, before the *n* + 1th track measurement, AI-based data is generated for . Data generation is an immediate response to avoid time delay when supplying recent track measurements for an assessment of track condition. This action, in turn, reduces the probability of dealing with track geometry-related unplanned maintenance due to late defect detection on the affected track code. For data generation purpose, an identified track geometrical parameter is defined as an autoregressive variable in a NARX model and is denoted as ${\widetilde{ y}}_{k}^{n+1}\left({x}_{i}^{{S}_{j}}\right)$. The proposed NARX model also takes external variables ${u}_{l}\left({x}_{i}^{{S}_{j}}\right)$ for as model inputs. Therefore, the corresponding NARX can be expressed as

1 $${\widetilde{y}}_{k}^{n+1}\left({x}_{i}^{{S}_{j}}\right)=f\left(\begin{array}{c}\begin{array}{c}{\widetilde{y}}_{k}^{n+1}\left({x}_{i-1}^{{S}_{j}}\right),{\widetilde{y}}_{k}^{n+1}\left({x}_{i-2}^{{S}_{j}}\right)\end{array},\dots ,{\widetilde{y}}_{k}^{n+1}\left({x}_{i-{d}_{1}}^{{S}_{j}}\right),\\ {u}_{1}\left({x}_{i-1}^{{S}_{j}}\right),{u}_{1}\left({x}_{i-2}^{{S}_{j}}\right),\dots ,{u}_{1}\left({x}_{i-{d}_{2}}^{{S}_{j}}\right),\\ {u}_{p}\left({x}_{i-1}^{{S}_{j}}\right),{u}_{p}\left({x}_{i-2}^{{S}_{j}}\right),\dots ,{u}_{p}\left({x}_{i-{d}_{2}}^{{S}_{j}}\right),\\ {u}_{p+1}\left({x}_{i-1}^{{S}_{j}}\right),{u}_{p+1}\left({x}_{i-2}^{{S}_{j}}\right),\dots ,{u}_{p+1}\left({x}_{i-{d}_{2}}^{{S}_{j}}\right)\end{array}\right)$$

where$f\left(\cdot \right)$ is an unknown nonlinear mapping function of $d={d}_{1}+\left(p+1\right){d}_{2}$’s previous known outputs. Each of the *p* external variables is defined by . At this point, track geometry parameter *n* is assumed to be irregular, and in terms of the targeted track geometry, *k* is non-independent index. The trade-off analysis between model complexity and model performance at the end of the model development process is used to justify these assumptions.

Generally, any external variable can be excluded from the NARX model if its contribution to the model performance comes at the expense of reducing model simplicity. On the other hand, the term '1' is added to account for the current size of change in the specified track geometrical parameter. The amount creates the difference between ${y}_{k}^{n}\left({x}_{i}^{{S}_{j}}\right)$ and ${y}_{k}^{n-1}\left({x}_{i}^{{S}_{j}}\right)$.

### Application of neural network on the NARX model

A neural network is a computational paradigm inspired by the structure of biological neural networks and their way of encoding and solving problems. The fundamental NN model is adjusted according to a user’s motive and resources available and is trained systematically with filtered input–output data to solve the problem under investigation.

**Figure 1:**
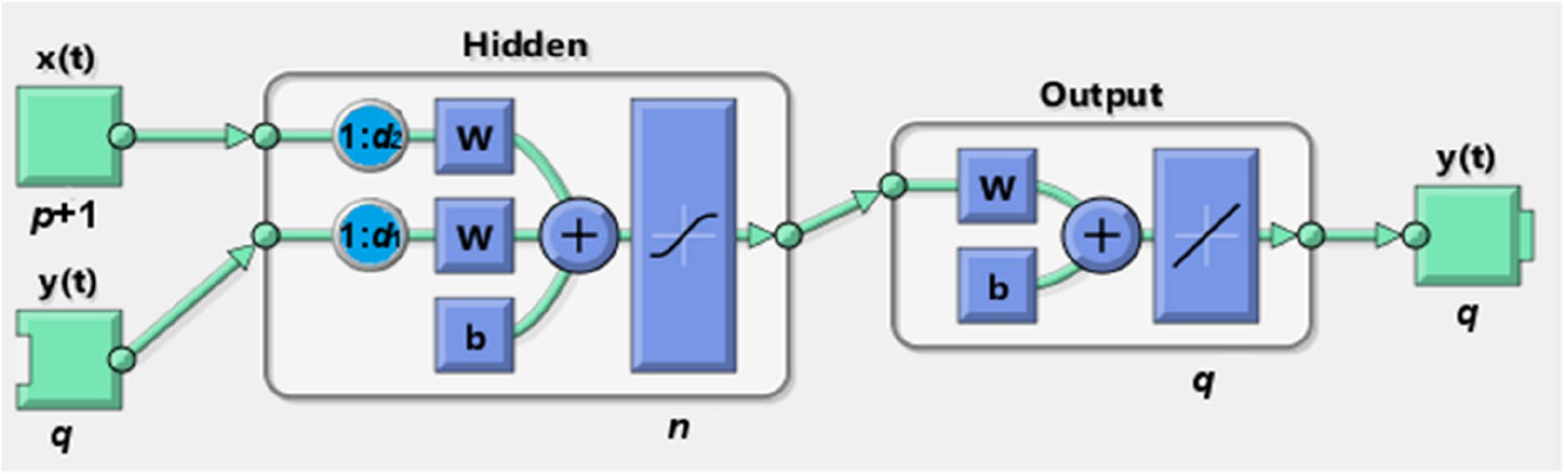
An overview of NARXNN architecture.

In this study, a NN is applied to obtain the nonlinear function $f\left(\cdot \right)$ in (1). This requires an appropriate network configuration in line with the model description. The NARX-NN architecture in Fig. 1 shows an input layer including *q* recurrent unit with *p* + 1 neurons, a hidden layer and an output layer with *q* neurons describing the dynamic relationship between input and output variables in (1). The lines represent weighted connections, and the squares represent bias thresholding nodes. A two-line circle in the hidden layer is the lag/delay element for an input variable. Here, the exact value of both *d*_1_ and *d*_2_ is unknown and will be determined based on model training result. NARX-NN has capability to deal with nonlinear inputs, which can be time-dependent (such as disruptions). This capability enables a better prediction compared with traditional neural networks, especially when input features are dynamic.

Prior to the NN training, a configuration of the network must be attained. The number of nodes in the input and output layers, respectively, reflects the number of input and output variable(s) of the system of interest. In case of a NARX-NN model, a user also has to initiate the value of a delay unit in model output and external series. Apart from that, the number of hidden nodes is generally unknown and would be iteratively adjusted during the network training. An independent test will later be conducted to assess an optimal architecture of NARX-NN model.

### Mitigation of risk of over fitting when using NN

When an NN is trained using a small training data set, the resulting NN-based model may perform poorly in any instances not included in the training set. The NN is likely over fitted to the training data, significantly reducing the generalisability of the model. Also, the network model tends to generate large errors when new instances are presented. Applying this limitation to data generation, the proposed NARX-NN might have a limited capacity to identify sudden shifts in the evolution of track condition over accumulated tonnage. To address this issue and improve model generalisation, a regularisation method is adopted in NN model training. Aside from demonstrating good generalisation properties, training the NN using the regularisation method is less time consuming, as a model validation step is unnecessary^13,18^. With a validation process scaled as O(${N}^{2}$) where *N* is the size of data, the benefits of the regularisation method become apparent in cases of large data sets^13^.

To obtain the best combination of weighting and bias values, producing a prediction model that generalises well, the network is trained on the selected architecture and training set to optimise the following performance function:

2 $$wMSE = \gamma E_{W} + \left( {1 - \gamma } \right)E_{D}$$

where $\gamma$ is the regularization parameter. ${E}_{W}$ and ${E}_{D}$ represent the mean of the sum of squares of the network weights and biases and the mean sum of squared of network errors, respectively. In the stated performance function that is a typical error function with an additional term, the training forces the NN to utilise small weights and biases, i.e., low ${E}_{W}$ value. Burden et al.^13^ and Piotrowski et al.^19^ stated that the successful application of NN to a problem requires a proper choice of learning algorithm; thus, the Bayesian regularisation (BR) technique is recommended if the data is small and prone to over fitting. Several assumptions must be made before any training when BR is adopted. The network weights and training data are considered random variables with a Gaussian prior distribution. The prior probability over the weight is then updated by incorporating training data according to Bayes’ rule. The optimal weights of the network are then determined when the posterior probability is maximised; this is equivalent to minimising the error function in (2).

Though NN can approximate any real-valued continuous functions its success is highly dependent upon a proper selection of NN’s components such as data preparation and pre-processing, network architecture, model assumptions and constraints, objective function and post-processing steps. In a particular case of disrupted TIS, this study has highlighted the importance of good NN configuration to model dynamics links between track geometrical parameters.

## Data

**Figure 2:**
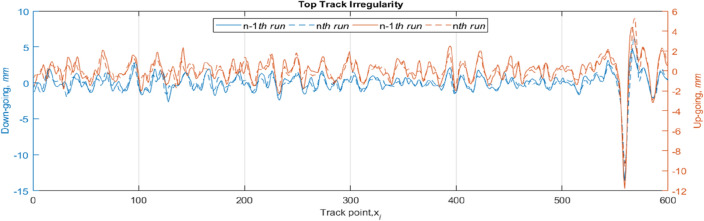
Two consecutive runs of track geometry inspection over the 300-m long track. Irregularity of the track is measured at every 0.5 m and this generates 600 track points as labelled in the position-axis. Note that this is a single set of temporal data across 300-m long track section.

Three segments of 100-m long track are identified and used to demonstrate how the proposed NARX-NN model should be operated during disruption. Track geometry data (including top, line, twists, and gauge) have been obtained for the specific track section over 20 consecutive years. The track geometry data has been measured and recorded by Track Inspection Vehicle and generally 4–5 routine inspections have been recorded annually. The geometry data is associated with the track condition indices (or called ‘track quality index’), spanning over 40 years of data records. Note that the segments are part of the particular track route’s routine inspections (e.g. > 200 km). Three track segments are attributed to the output of the non-overlapping segmentation algorithm applied to a collection of data series displayed in Fig. 2. The collection consists of two pairs of longitudinal level measurements recorded at two consecutive time points where each pair of data belongs to the same rail side of an analysed track segment. The notations *S1*, *S2,* and *S3* are used to differentiate the analysed segments. Due to limited permission given, this study cannot disclose the location of inspected track segments, source of inspection data as well as the data owner. For generalisation purpose, the *x*-axis in the Fig. 2 is marked with a series of integer; starts with 1 and ends at 600 (= 300*r).

**Figure 3:**
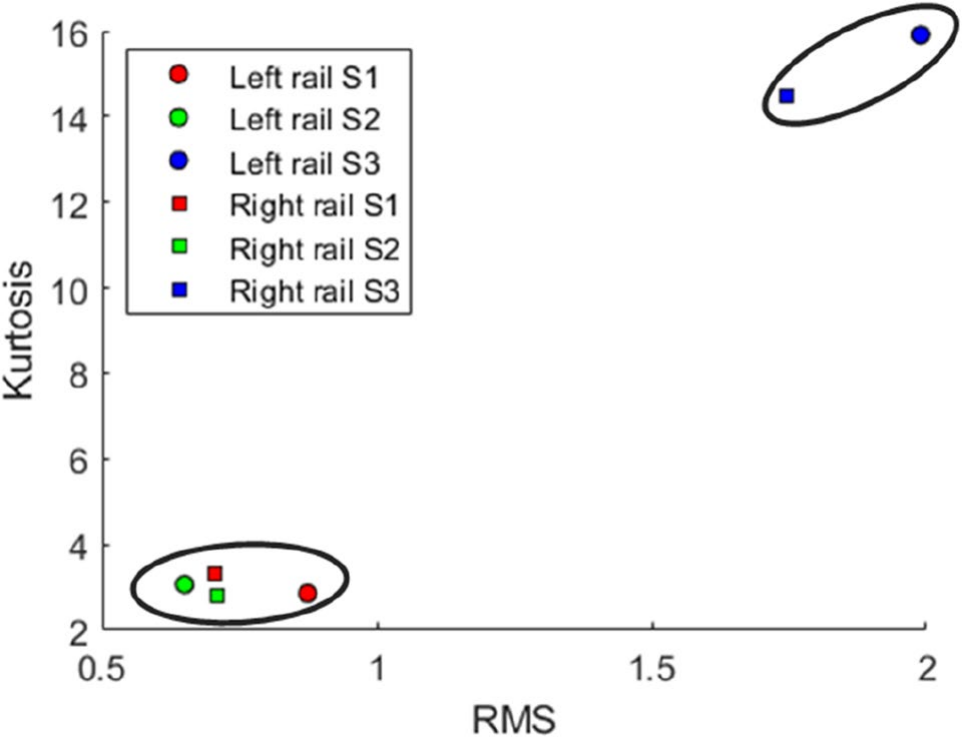
Application of distance-based clustering at the pre-processing step.

Visual observations of the data series in *S1* and *S2* allowed for the hypothesis that the two data series might have a high degree of similarity in terms of statistical characteristics. If evidence corroborates this trait, one of the data series should be removed before entering the NN training stage to avoid presenting redundant and unproductive findings with this study. To investigate the removal decision, the visual evidence was processed using the scatter plot shown in Fig. 3. In this figure, a plot of RMS versus the kurtosis of data series for each track segment is built. RMS and kurtosis are widely-used statistical indicators used in signal characteristic analysis^20^. The resulting clusters seen in Fig. 3 support the hypothesis; thus, to select which series to remove, the Euclidean distance between the left and right points (rail) in the scatter plot is used as a tie-breaker. *S2* is then selected to join *S3* in NN learning based on its wider distance.

## Results and discussion

To demonstrate the effectiveness of the proposed data generation model, we have selected a track longitudinal level to act as the disrupted track measurement, which is later referred to as a research object. A track’s longitudinal level is the vertical deviation of the upper surface of the head of the rail (running table) from the mean vertical position (reference line). Longitudinal levelling is among many track geometrical parameters measured by a track recording car and has been acknowledged as the leading indicator for track tamping maintenance decisions^21^. This establishment is due to the fact that track irregularities in the vertical direction (or called ‘top’) evolve faster than other track geometry defects. Note that the longitudinal level is assessed for both left and right rails and this requirement has imposed two nodes in the output layer of our NARX-NN model.

To unify with the neural network's terminology, we will use input/output data series term to denote track geometrical parameter over an analysed track segment applied in the data generation model. The primary motivation for the use of external data series in the proposed data generation model is stemmed from unparalleled characteristics of the response action we seek for a disrupted track measurement. Data integration was performed to the past and present measurement values of other track geometrical parameters, e.g., alignment and gauge, together with the past values of the research object to forecast the size the research object would evolve in both spatial and time for a short period of service time. A fusion method of integrated NARX and neural network (denoted as NARX-NN) is expected to reveal nonlinear relations between these geometrical parameters over a span track length whose variations in track stiffness are undisclosed.

A rationale behind the use of track alignment in the NARX-NN model is its influences over vertical track forces as well as the longitudinal level^22^. A longitudinal track level and track alignment have been combined in various ways to manage track geometry problems^23^. For example, Soleimanmeigouni et al.^24^ established such level of relationship between these parameters in the long-term track deterioration process. A similar relationship might exist in the short-term prediction model, but direct use of this relationship might be inappropriate due to a different set of model constraints. A key distinction between short and long term track prediction model is the validity of track measurements (or track quality indices) on the aspect of track deterioration where models in the latter category describes the gradual deterioration of track whereas a sudden shift/abrupt changes in track deterioration is the former model's focus^16,25^. For short-term prediction model, the tamping effect is only valid to the first track measurement after a maintenance work covering the analysed track segment. Nevertheless, nonlinear interactions between track alignment and longitudinal level would be worthwhile to be investigated on the context of disruption management. Similar to longitudinal level, a deviation of track alignment is also measured and analysed for left and right rails simultaneously. Apart from track geometries, track gauge measurement is used as an exogenous variable in our NARX model. Variations in track geometries and gauge can lead to large lateral wheel and axle forces, resulting in derailment or damage to the structure of the track^26^.

As explained earlier, the prediction model in this study has been built after the completion of *n* successive track measurement runs. This mechanism means that *n* previous track measurements are accessible. Hence, a series of deterioration rates of a longitudinal level is defined as the final external variable for the NARX-NN model.

### Network training

**Figure 4:**
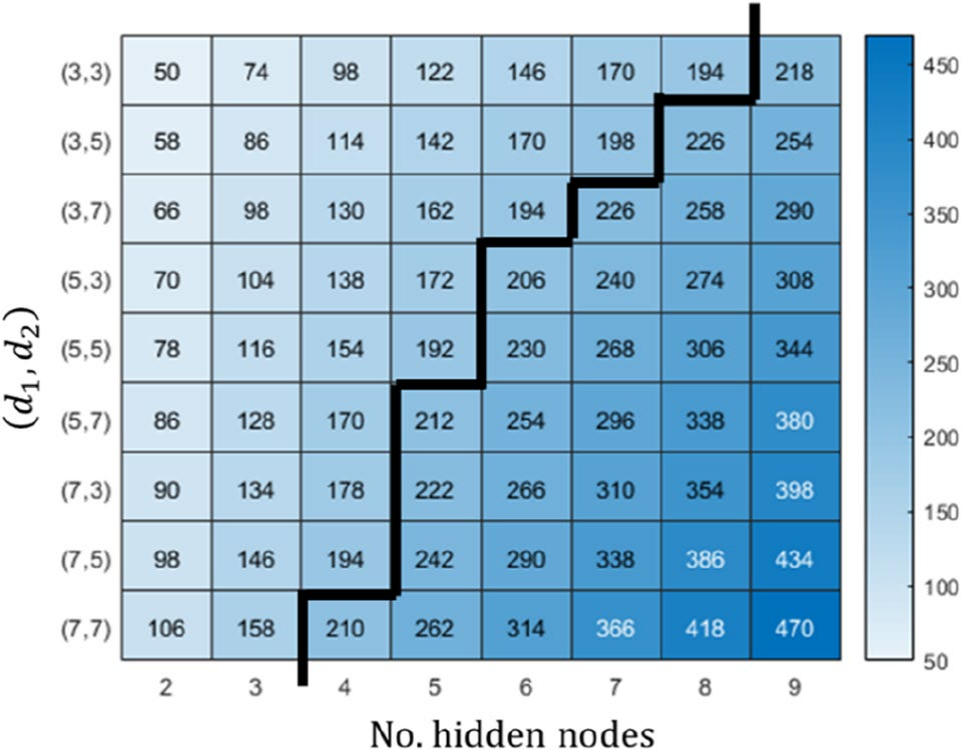
Degree of freedom of NARX-NN for a designated boundary condition imposed on delay units; *d*_1_ and *d*_2_. The number of hidden nodes of the network is restricted to nine corresponds to *N* = 200.

Training NN is a data-intensive task and there always possibility NN over fit data, which cause, for example, the resulted prediction model does a poor job predicting new data. An effective way to mitigate the risk of over fitting in NN-based function approximation is by controlling complexity in a network, i.e. avoid oversized network. A simple decision rule based on a degree of freedom in a NN model is to keep the number of parameters in a network less than the size of training set^27^. In our case study, we discretise the track route into elemental track sections, each with 100 m length (or *N* = 200). When the discretised size of the training set is certain, *N* = 200 (200 spatial data point for a specific track section of 100 m long), a mapping technique is applied on the set to determine feasible selection(s) of network topology in this study. The notation NARX-NN $\left({d}_{1},{d}_{2},n\right)$ represents a network topology for our NARX-NN model will be used in the remaining texts. An example of mapping output is given in Fig. 4 where an integer in each cell represents the number of network parameter in a NARX-NN. Based on the decision rule, only NARX-NN models on the left side of the boundary line (marked with a bold black line) in Fig. 4 are trained to find the best network.

Selected networks from the previously filtering step are trained with a collection of data series associated with *S2* and *S3*. During the training stage, each model received 30 repetitions of simulations with random initialization.

**Figure 5:**
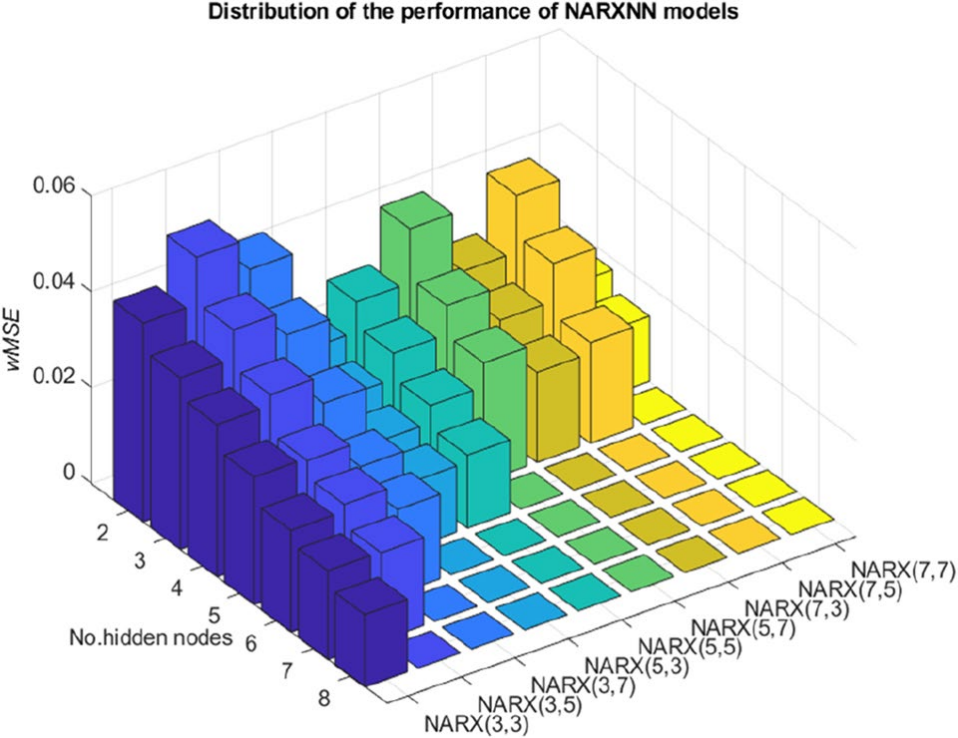
Mean values of NN training results for a range selection of network topology.

The average values of the weighted MSE (*w*MSE) over 30 samples are recorded in Fig. 5 for all networks. First of all, for each network, as the number of hidden nodes increases the *w*MSE start to decrease, i.e., increases in model performance. The evolution of *w*MSE over *x*-axis occurs steadily in each network. This observation encourages us to further test networks with a large number of hidden nodes.

On the other hand, regardless of the number of hidden nodes configured in the NARX-NN $\left({d}_{1},{d}_{2},n\right)$, we can observe a clear pattern about the effect of the parameter ${d}_{2}$ in the performance of the network. For instance, for networks under NARX-NN $\left({d}_{1}=3,{d}_{2},n\right)$, the *w*MSE is substantially increased when a network in this group takes additional delay units in the external variables i.e.${d}_{2}>{d}_{1}$. The same pattern also appears for NARX-NN $\left(5,{d}_{2},n\right)$. However, NARX-NN $\left(7,{d}_{2},n\right)$ experiences an increment in the *w*MSE when ${d}_{2}<{d}_{1}$. These two opposite observations highlight the existence of the mixed effects of external variables on the performance of our NARX model. In other words, information between adjacent track points is not necessarily meaningful to accurately predict 'missing' longitudinal level.

### Independent test

**Figure 6:**
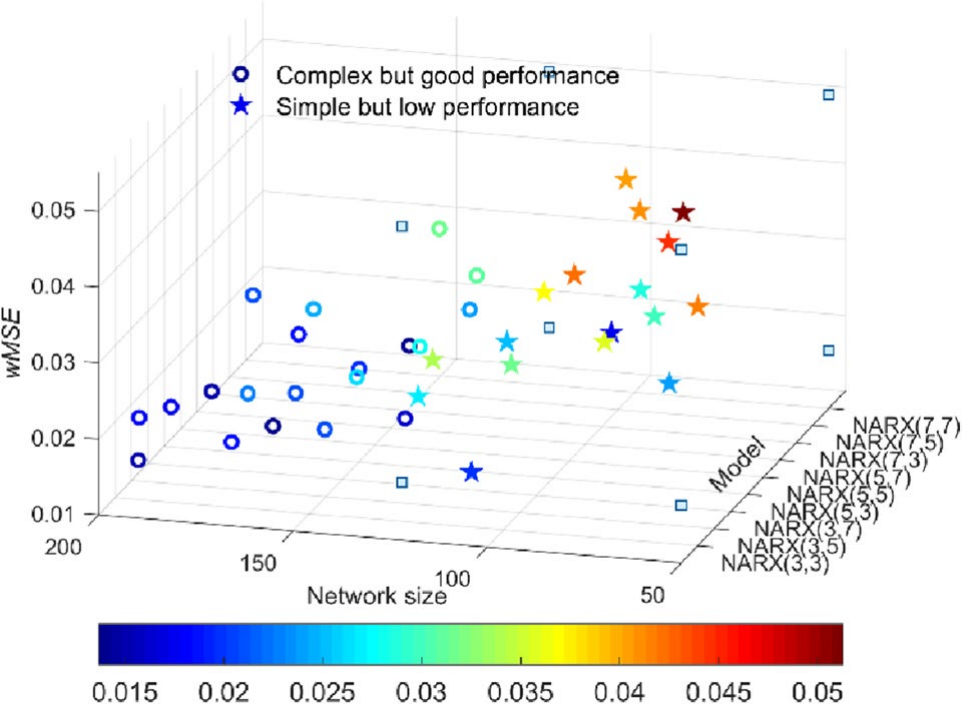
The appearance of *k* = 2 clusters of NARX-NN models for S2 case. Resulting clusters indicate that any networks with a number of parameters less than 150 should be considered as a simple prediction model.

A large number of NNs have been developed with varying numbers of hidden layers, nodes and neurons. The inputs and outputs are integers. Among all trained networks, we have selected NARX-NN(3,3,8), NARX-NN(5,3,5) and NARX-NN(7,7,3), showing the best predictions, for an independent test. These networks belong to the 'complex but good performance' cluster where the complete clustering results on all networks are presented in Fig. 6. The k-means technique was used for clustering which was performed subject to a pairwise value of (model simplicity, *w*MSE).

**Figure 7:**
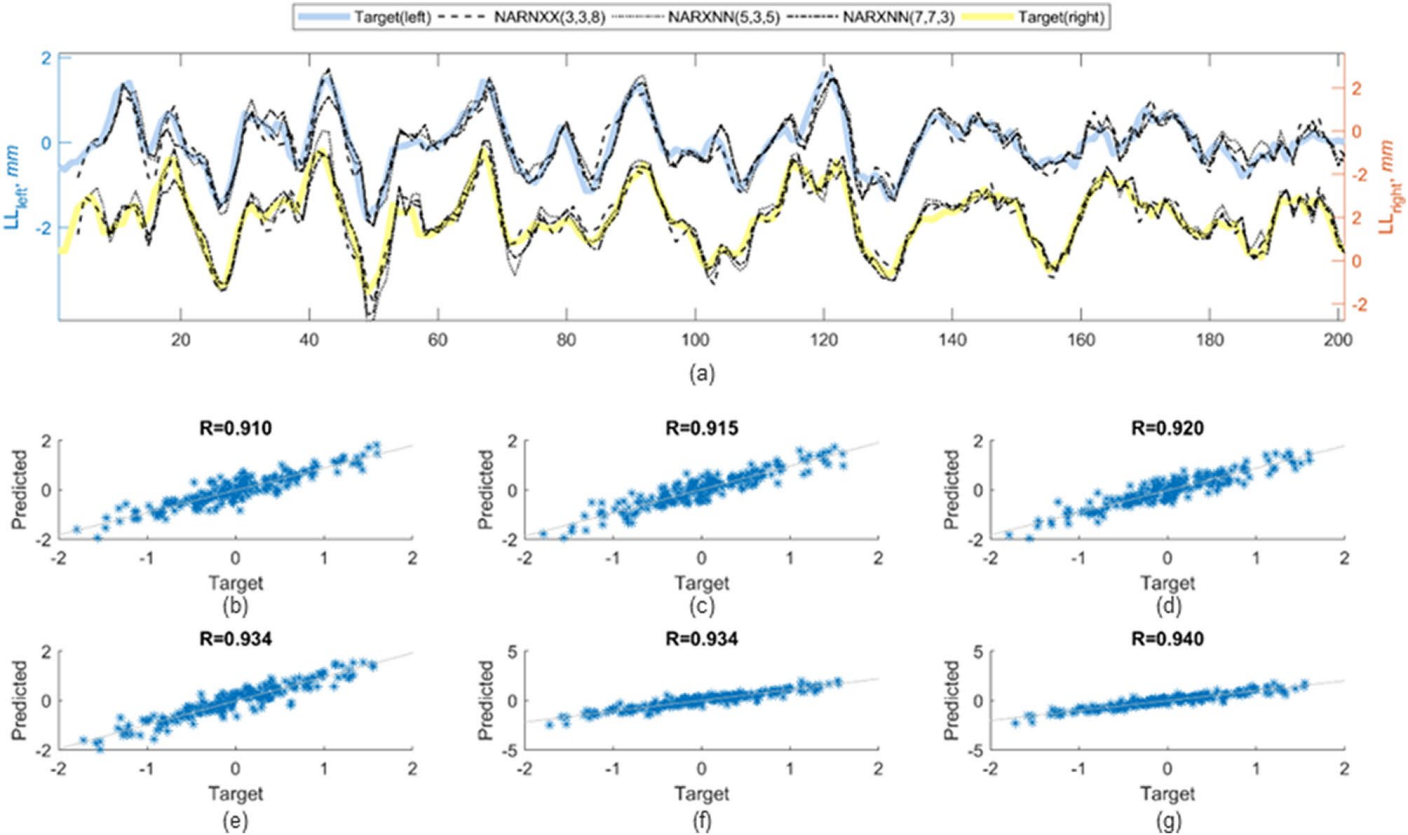
Predicted and target values of longitudinal track level of *S2* for left and right rail are superimposed in the plot (**a**). Overall prediction accuracy of different networks can be observed from the scatter plot (**b**–**d**) and (**e**–**g**) for left and right rail, respectively.

Prior to the independent tests, the test data series formed by the *n* + *1*th track measurements are split into two parts. The first part contained track measurements data from the first *d*_*3*_ track points; this has been reserved for network initialization. The value of *d*_*3*_ will vary from one network to another, as it takes the largest of *d*_*1*_ and *d*_*2*_ within the network under investigation. The remaining part of the test data series is later compared with the response from the network outputs. Figure 7a shows the predicted value of longitudinal level for ${x}_{i}$ in the range of $\left[{d}_{3},m\right]$ for all tested networks. Observations from the correlation plots in Fig. 7b–g show that the three models predicted longitudinal level correctly based on the high correlation between predicted and target values.

**Figure 8:**
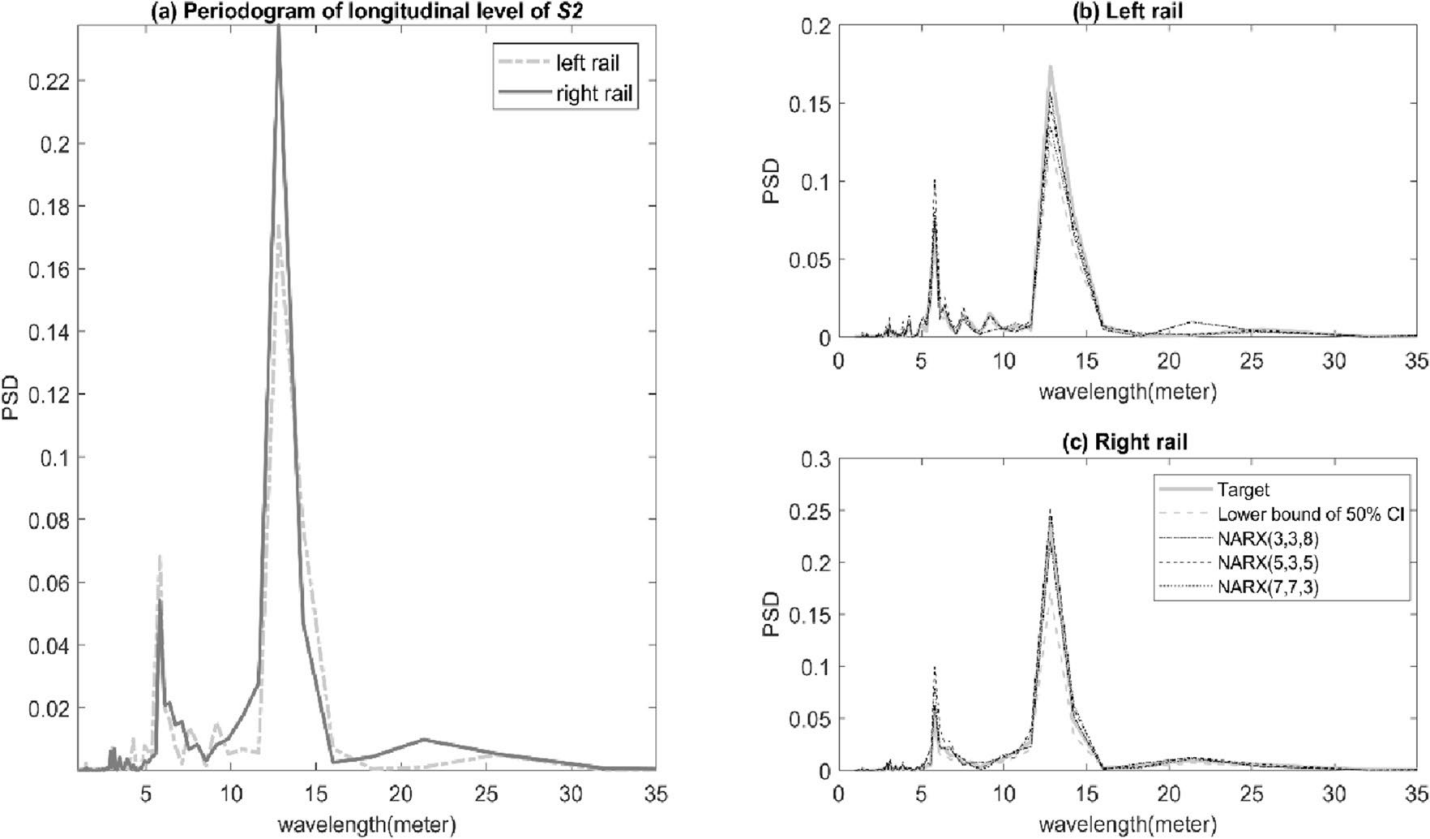
PSD comparisons between (**a**) the left and right rail, and artificial and actual longitudinal track level of (**b**) left rail, and (**c**) right rail for S2.

Unique characteristics of track geometry measurements can be defined in terms of power spectral density (PSD). PSD is a tool often used for spectral analysis. Here, the PSD was used to examine the relation between the artificial and actual longitudinal level data. In Fig. 8a, the wavelengths of the actual longitudinal level for both left and right rail are included between ~ 3 and ~ 35 m. Track-geometry related defects in this wavelength range are characterised as a reliable indicator of track maintenance (e.g. tamping and resurfacing)^28^. Peaks of PSD for left and right rail were most apparent the wavelength of ~ 7 and ~ 13 m. However, a peak of PSD at the ~ 13 m wavelength of the right rail has been found approximately 35% higher than the left rail. This would indicate that the condition of the left rail is relatively better than the right rail. When the outputs of NARX-NN model have been evaluated in the frequency domain, we have observed the results in Fig. 8b and c that show high and positive correlations between the AI-based and actual track longitudinal level. Also, there are no statistically significant differences (tested on a narrow confidence interval) in the PSD’s values of the predicted and actual longitudinal level at identified distinct peaks.

**Figure 9:**
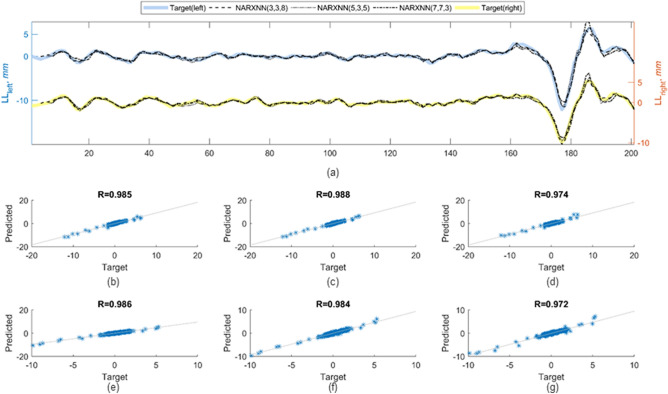
Predicted and target values of longitudinal track level of S3 for left and right rail are superimposed in the plot (**a**). Overall prediction accuracy of different networks can be observed from the scatter plot (**b**–**d**) and (**e**–**g**) for left and right rail, respectively.

**Figure 10:**
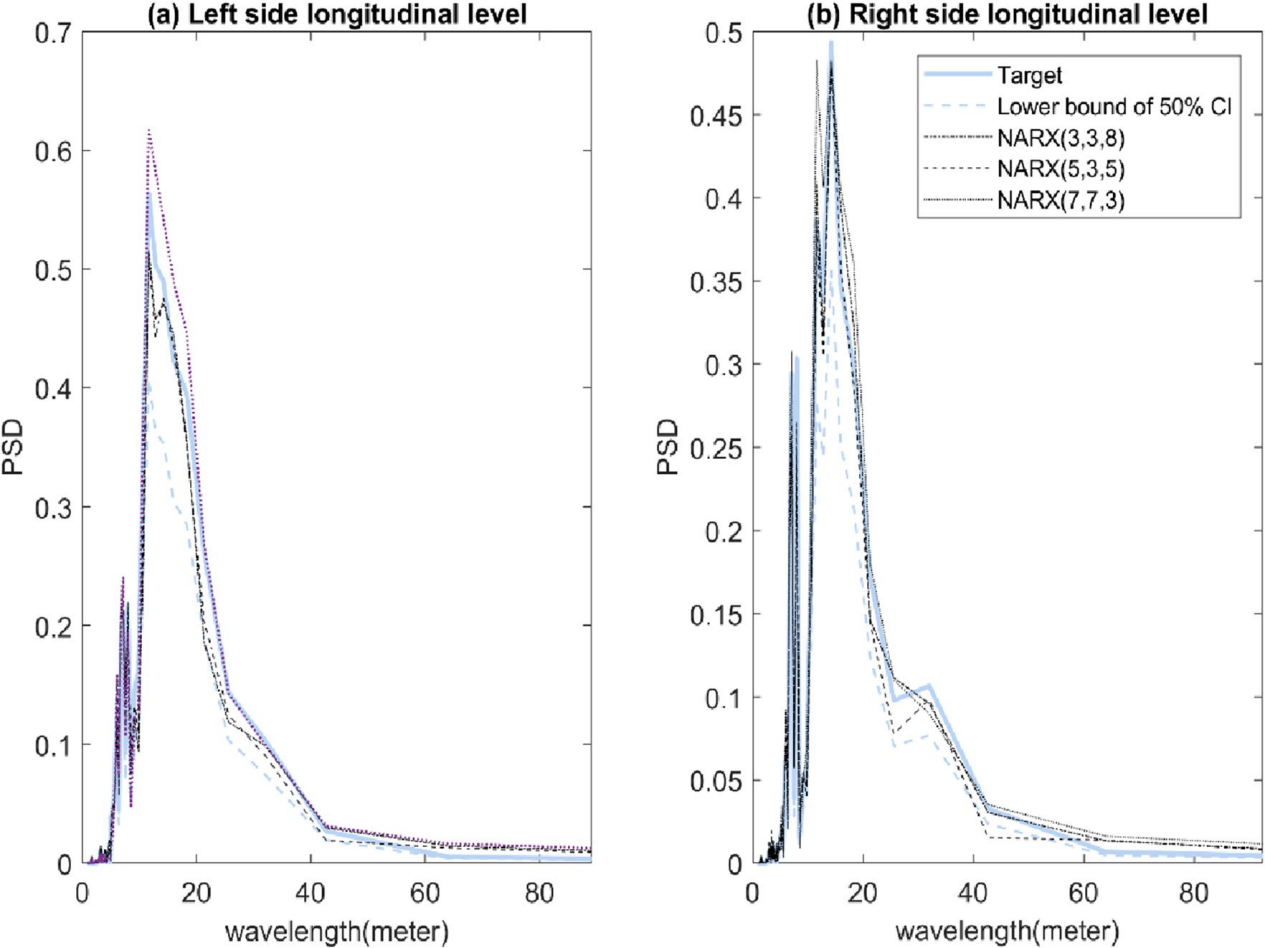
PSD comparison between artificial and actual longitudinal track level of (**a**) left rail, and (**b**) right rail for S3.

As demonstrated earlier, the three selected NARX networks successfully predict the ‘missing’ longitudinal level (left and right) for *S2*. To investigate whether the same networks could be applied directly to another track segment, an independent test has been performed on *S3*. Based on the results of the goodness-of-fit test presented in Fig. 9, we have demonstrated that each of the selected NARX networks exhibits good generalisation ability. Additional merit to this claim is provided in Fig. 10. It is evident in the figure the establishment of positive correlation in terms of the wavelength of pronounced PSD’s peaks between the AI-based and actual data for the left and right rail.

### Discussion

The NARX-NN model exhibits promising generalisation features supported by excellent prediction results from the single NARX-NN model as used on two weak-relation test sets; *S2* and *S3*. These new findings highlight that a pre-processing step to categorize a profile of track substructure, e.g., fouled ballast modulus, for the unit track sections tested is not necessarily required for the prediction model to perform well. However, a better prediction result may exist if a dedicated model for a specific class of unit track section is employed.

For example, track classes (low, medium or high) can be established based on the degree of relationship between longitudinal track level and the moisture susceptibility of ballast/sub ballast layer^29^. Results of this study reveal that special track areas like those in transition zones with high moisture in the ballast and sub-ballast layers tend to experience significant deterioration of longitudinal track level. Therefore, it is possible to redefine the length of the track segment as well as its splitting points. Different mechanisms of track data segmentation can be found in Soleimanmeigouni et al.^30^.

An analysis of track measurement in the wavelength (or frequency) domain is useful when information about the shape of track defects and their wavelength content is needed^23^. The information is further processed to describe track quality condition^31,32^. When considering spectral analyses, the PSD of track measurement is calculated where the corresponding power spectrums graph such as in Figs. 8 and 10 provide quality indicators to an analysed track segment. Results from both figures show that AI-based data well replicates the spectral components of the actual data. This is a solid foundation to build on AI-based data to describe the current state of track integrity. Previous studies such as Berawi^31^ illustrates and demonstrates how statistical information of PSD can be used to assess track condition. For example, periodic peaks in PSD at specific wavelength might indicate excessive deterioration of ballast or effects induced during rail strengthening process^31^. Thus, the use of AI-based data in an assessment of track condition would not introduce bias in the process of track maintenance decision. Finally, the novel findings and results in this study also suggest that the proposed NARX-NN model can be re-configured to generate data for other track geometrical parameters.

## Conclusion

Dealing with low probability events demands for (special) innovation in affordable risk response strategies. Fortunately, an affordable but reliable strategy can be developed from a smart partnership between artificial intelligence and temporal-spatial data analysis. This study aims at developing a data generation model for disrupted track measurements in the event of disrupted TRC operation. The joint application of machine learning, NARX and recurrent NN in the model development is very successful based on the impressive results of correlation analysis and PSD comparison as seen in Figs. 7, 8, 9 and 10.

An initial design of the proposed NARX-NN model has been tailored to respond to disruptive events in a planned inspection schedule. This specification makes the model is useful for a specific length of time-step which is derived from an inspection interval. Though an interpolation can be applied to generate artificial data for smaller time-step it can only be performed outside the NARX-NN model. This approach excludes dynamic interactions between input/output variables and error dependencies in the results if some simulations of abrupt transitions in a deterioration of track condition within a short time period are required. Thus, future works should focus on equipping the existing NARX-NN model with baseline-point estimation with anomalies for any time instant between two inspection runs. Future work will also emphasise on the field implementation of our NARX-NN model.

## Data Availability

The datasets used and/or analysed during the current study available from the corresponding author on reasonable request.
